# Extracellular Phosphate Modulation and Polyphosphate Accumulation by *Corynebacterium matruchotii* and *Streptococcus mutans*

**DOI:** 10.3390/dj12110366

**Published:** 2024-11-16

**Authors:** Debarati Ghose, Robert S. Jones

**Affiliations:** 1 Department of Biology, Texas A&M University, College Station, TX 77840, USA; dghose@bio.tamu.edu; 2 Department of Developmental & Surgical Sciences, University of Minnesota, Minneapolis, MN 55455, USA

**Keywords:** oral, bacteria, calcium, phosphate, polyphosphate

## Abstract

(1) Background: An alternative and understudied microbial mechanism that may influence demineralization is the microbially mediated ion exchange of Ca^2+^ and orthophosphate (P_i_), which alters the saturation state of the mineral species within the surface enamel. There is a need to examine the ability of members of the oral microbiome to modulate Ca^2+^ and P_i_, which control mineral solubility, in order to effectively evaluate mineralization therapies to improve oral health. (2) Methods: P_i_ uptake was measured using an ascorbic acid assay during a BHI liquid culture growth of *Corynebacterium matruchotii* and *Streptococcus mutans* for up to 20 h. The initial and endpoint medium Ca^2+^ levels were measured using ICP-OES. Bacterial cells were examined at different growth stages using DAPI/polyP binding emission at 525 nm to detect the presence of internalized macromolecules of polyphosphates (polyP) that could drive P_i_ uptake. (3) Results: *C. matruchotii* (*p* = 0.0061) substantially accumulated P_i_ (3.84 mmol/L), with a concomitant formation of polyP. In contrast, *S. mutans did* not take up P_i_ or accumulate polyP. No significant Ca^2+^ drawdown in the media was observed in either strain. (4) Conclusions: This study suggests that when examining the future efficacy of prevention technologies to improve, in vitro assays may consider including specific oral bacteria capable of substantial P_i_ uptake.

## 1. Introduction

An understudied mechanism that may affect the efficacy of caries inhibition of bioactive dental therapies and biomaterials is the uptake of orthophosphate (PO_4_^3−^, P_i_) and the subsequent synthesis of long-chain polyanion polyphosphate molecules (polyP) by bacteria [1,2]. PolyP can also chelate cations, such as Ca^2+^ [3,4]. The lack of investigation into the uptake of these ions in the oral environment may be attributed to several decades of virulence factors, such as pH modulation, acid tolerance, and biofilm adherence [5,6,7]. With the introduction of the plaque ecology hypothesis, researchers have expanded caries investigation beyond traditional single-species models [8,9,10]. Recent advancements in 16S rRNA gene sequencing have allowed for the investigation of caries susceptibility in the context of the broader ecology of bacteria [11,12,13]. Microbiome studies have confirmed the complexity of assigning microbial taxa and their relative abundances to definitive oral health status [14,15,16,17,18,19,20,21]. These studies also question the validity of defining bacteria as commensal or pathogenic because the phenotypic behaviors of these bacteria can vary drastically depending on the dietary and environmental factors influencing the biofilm communities that constitute dental plaque.

Many classically defined commensal bacteria have clear associations with decreasing dental enamel solubility through their ability to either compete directly with acidogenic bacteria or mitigate acidic pH challenges [22,23,24,25,26]. However, many bacteria which have been viewed as commensal readily ferment carbohydrates through glycolytic pathways that produce acidic conditions similar to those produced by traditionally defined pathogens [27]. Thus, acidity is not the only factor contributing to the risk of caries and enamel demineralization. An alternative and understudied microbial mechanism that may influence demineralization is the microbially mediated ion exchange of Ca^2+^ and P_i_, which alters the saturation state of the mineral species within the surface enamel. Importantly, exogenous topical therapies and bioactive restoration can mitigate such demineralization.

Dental enamel is composed of different forms of hydroxyapatite (HA) with varying ionic substitutions and solubilities. The solubility of HA is based on the activity/concentration of the constituent ions in the solution near the crystal surface. Microbially generated acids directly interact with solubilized (albeit at low concentrations) P_i_ and OH^−^ near the tooth interface. When the acid reacts with these ions and changes their activity/concentration, there is a shift in the localized saturation index (ion-dependent solubility) of HA towards demineralization [28]. A similar effect to this pH modulation may be achieved by directly changing the activity/concentration of Ca^2+^ and P_i_ within the aqueous phase at the tooth interface. This could potentially be achieved by internalizing these ions inside the bacterial cells near the tooth interface, which could reduce their effective activity/concentration with respect to tooth mineral solubility [1].

It has been postulated that oral bacteria may produce undersaturated conditions for HA through the extracellular binding of Ca^2+^ and P_i_ [29,30,31] or through the incorporation of these ions into cellular material [32]. Our group has recently demonstrated that two oral bacteria, *Lactobacillus rhamnosus* and *Rothia dentocariosa*, have a similar mechanism of phosphate uptake via polyP synthesis to that found in marine environments [1,2]. In our previous work, we investigated annotated genomes within the DOE’s Integrated Microbial Genome [33] pipeline from an initial list of 1392 microbial genomes of oral taxa within the Human Oral Microbiome Database (HOMD) [34]. This genomic analysis revealed that some oral bacterial clades contain genes known to code for enzymes responsible for synthesizing polyP (polyphosphate kinases *ppk1*, and *ppk2*), as well as for enzymes that may subsequently hydrolyze polyP (exopolyphosphatase *ppx*).

In this study, the temporal patterns of P_i_ and Ca^2+^ uptake, along with polyP imaging, were assessed across different stages of growth for a commensal bacterial strain and a caries-associated bacterial strain. *Corynebacterium matruchotii* (ATCC 14266) was used in this study to represent a classically defined commensal bacterial strain with the genetic potential to accumulate polyP. *Streptococcus mutans* (ATCC 700610) was used as the classically defined caries pathogen [35,36], but it does not possess the canonical polyP biosynthetic genes. The objective of this work was to measure the uptake of Pi and Ca^2+^ by a canonical caries-associated bacterial strain that lacks polyP-associated genes (*Streptococcus mutans*) and to compare this uptake with that of a bacterial strain that possesses common polyP metabolic genes (*Corynebacterium matruchotii*).

## 2. Materials and Methods

### 2.1. Cultivation

The cultures used in this study were regularly stored in glycerated Brain–Heart Infusion (BHI, 50%) broth at −80 °C and grown on BHI agar plates and broth as needed. *C. matruchotii* (ATCC 14266) was grown at 37 °C aerobically under shaking conditions. *S. mutans* (ATCC 700610) was grown anaerobically at 37 °C without shaking in an anaerobic chamber (Coy Laboratory Products, Grass Lake, MI, USA) with an atmosphere of 5% CO_2_ and 95% N_2_. For the experiments described herein, fresh overnight seed cultures of each bacterium were inoculated into 3 flasks, each containing 100 mL of BHI medium. Each flask was subsampled every hour for pH, optical density, P_i_ uptake, and visualization of polyP inclusions. Optical density (OD) at 600 nm was measured using a Genesys 50 UV–Visible Spectrophotometer (ThermoScientific, Waltham, MA, USA).

### 2.2. Phosphate and Calcium Uptake Quantification

**Figure 1:**
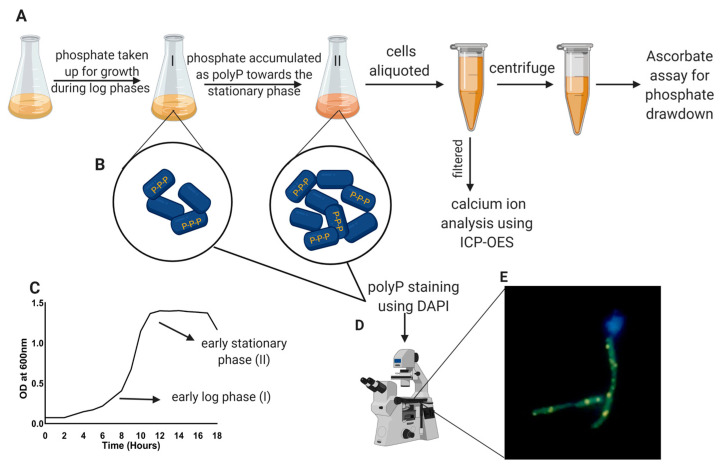
A schematic diagram of the experimental workflow. (**A**). Sterile BHI was inoculated with fresh seed culture, and growth was monitored hourly at 600 nm. Over time, the concentration of available soluble orthophosphate in the medium decreases as the bacteria take up phosphate and accumulate it as polyP. Cells were aliquoted at regular intervals during growth and one aliquot was centrifuged. The supernatant was used for an ascorbate assay to determine phosphate drawdown. The second aliquot was filtered, and the filtrate was used for the analysis of calcium drawdown from the medium using ICP-OES. (**B**). The two circles show bacterial cells at an early log phase and at an early stationary phase, respectively. PolyP accumulation is significantly increased as cells transition from the exponential log phase to the stationary phase (shown as yellow chains of polyP inside blue cells). (**C**). A typical bacterial growth curve; arrows indicate the growth phases (I = early log phase and II = early stationary phase) where cells were aliquoted for polyP study. (**D**). Cells aliquoted at both the early log and early stationary phases were stained with DAPI and imaged using an inverted fluorescence microscope for the qualitative identification of polyP. (**E**). Shows a typical epifluorescence image of *C. matruchotii* with accumulated polyP inclusions (yellowish green).

The bacterial uptake of inorganic orthophosphate (P_i_) from the medium was quantitatively measured using a modified ascorbic acid assay [37]. Aliquots (1 mL) were collected between the early exponential log and stationary phases and centrifuged at 10,000× *g* for 10 min at 4 °C. The supernatant was stored at 4 °C and then used for the P_i_ quantification assay (Figure 1A,B). A total of 100 µL of ascorbic acid/mixed reagent (ammonium heptamolybdate tetrahydrate and potassium antimony tartrate) was added to 1 ml of the cell-free extract. The assay was spectrophotometrically read at 880 nm for the quantification of inorganic phosphate (hydrolyzed form (PO_4_)^3−^ in case of polyP (PO_4_)_n_) (Epoch, Biotek, VT, USA). To complement our P_i_ uptake assay, the total Ca^2+^ uptake from the medium was measured by inductively coupled plasma optical emission spectrometry (ICP-OES) using an iCap 7600Duo ICP-OES Analyzer (ThermoFisher Scientific, Waltham, MA, USA). ICP-OES measurements were performed by the University of Minnesota Water and Soil Testing and Research Analytical Laboratory. For each replicate, 5 mL aliquots at the early log phase and early stationary phase were syringe-filtered using a 0.22 µm filter and the filtrate was used for Ca^2+^ quantification. To test for differences in ion concentrations between the early log phase and early stationary phase, a repeated measures ANOVA was performed for each bacterium, and the significance of differences are reported. Statistical analyses were performed using Medcalc (version 22.023, Ostend, Belgium).

### 2.3. Polyphosphate Visualization Using Fluorescence Microscopy

Cell pellets from the early log phase and early stationary phase (Figure 1C) were fixed in 1 mL of 50% ethanol to maintain the integrity of the accumulated polyP [1]. The fixed cells were stored at −20 °C for subsequent DAPI staining and the microscopic identification of polyP. A total of 40 µL of each replicate was air-dried onto a poly-L-lysine-coated well slide (Shandon^TM^ polysine slide, ThermoFisher Scientific, Waltham, MA, USA). A total of 40 µL DAPI (5 µg/mL) was added to each well and the plate was incubated in a hybridization chamber in the dark for 30 min. An Olympus IX81 fluorescence microscope fitted with an XM10 CCD camera and CellSens Dimensions Imaging Software (version 1.18 Build 16686) was used (Figure 1D). To visualize polyP inclusions, custom band-pass filters (Chroma Technologies, Bellows Falls, VT, USA) were used (DNA/DAPI excitation/emission (nm) 345/455 and polyP/DAPI excitation/emission (nm) 415/550). The fluorescent dye 4′, 6-diamidino-2-phenylindole (DAPI), when bound to DNA, has an emission spectrum at 456 nm (blue fluorescence). DAPI also binds to polyP, but the resulting polyP-DAPI complex shifts its emission to 525 nm (bright yellowish-green fluorescence) (Figure 1E). This difference can be exploited to differentiate polyP-DAPI complexes from DNA-DAPI [38] and to aid in the visualization of polyP granules synthesized by oral bacteria. Camera exposure and color levels were uniformly adjusted prior to sample imaging.

## 3. Results

### 3.1. Concentration Changes in Extracellular Orthophosphate and Calcium

**Figure 2:**
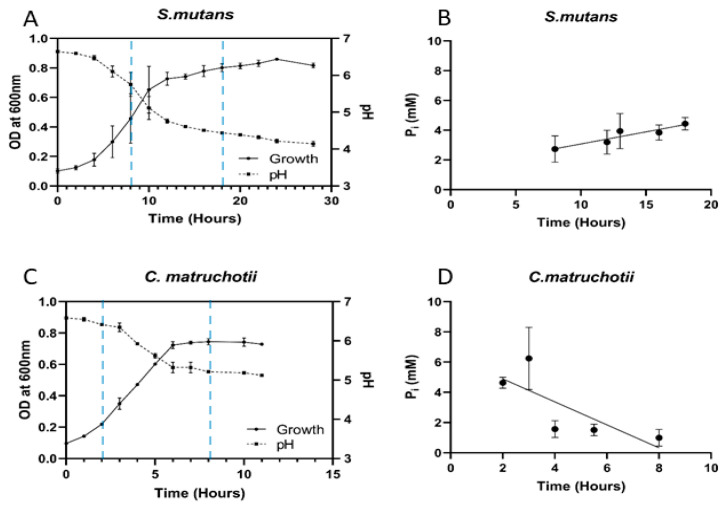
A comparison of the growth and pH changes in oral bacteria in BHI (left) and changes to P_i_ within the growth medium in the corresponding cultures (right). (**A**) + (**B**) = *S. mutans*; (**C**) + (**D**) = *C. matruchotii*; the time period in each growth curve, which corresponds to the time between early log and early stationary growth demarcated between the blue dashed lines, was used to assess changes to P_i_ within the growth medium. All growth studies and phosphate assays were performed in triplicate.

**Figure 3:**
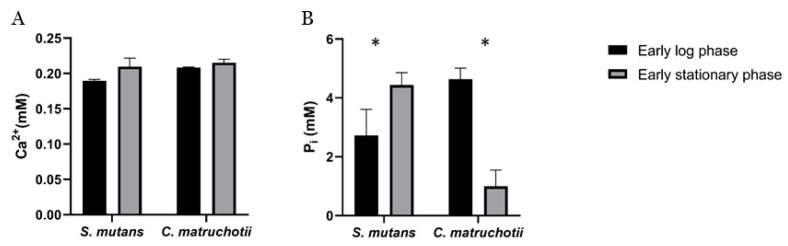
Changes in Ca^2+^ (**A**) and P_i_ (**B**) in *S. mutans* and *C. matruchotii* from an early log phase to an early stationary phase in BHI. Each data point was measured in triplicate. * Indicates significant differences in ion concentrations between an early log phase and an early stationary phase at *p* < 0.05.

Cultures of both *C. matruchotii* and *S. mutans* exhibited a decrease in pH, which correlated with an increase in OD; however, the two cultures differed in their influence on extracellular P_i_ concentrations (Figure 2A–D). Both bacteria showed similar overall growth yields, but the growth rate differed slightly between the strains (Figure 2A,C). Therefore, P_i_ uptake was assessed during the period between the early log and early stationary growth phases for each strain. A statistically significant (*p* = 0.0061) uptake of available P_i_ was observed in *C. matruchotii* (Figure 2D) but not in *S. mutans*. Instead, the available P_i_ concentration slightly increased (*p* = 0.0335) towards the stationary phase (Figure 2B). The changes in extracellular calcium concentrations between the early log phase and early stationary phase for both test strains are shown in (Figure 3A). Unlike changes in P_i_ (Figure 3B), no statistically significant changes in Ca^2+^ levels were observed between the early log phases and early stationary phases (Figure 3A).

### 3.2. Visualization of PolyP Inclusions

**Figure 4:**
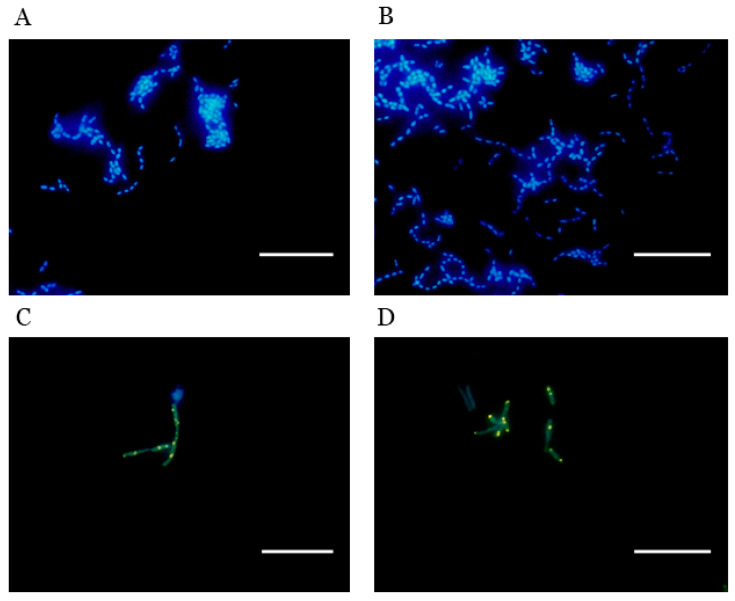
(**A**–**D**) Epifluorescent images of bacterial cells stained with DAPI; (**A**) + (**B**) = *S. mutans*; (**C**) + (**D**) = *C. matruchotii*; (**A**,**C**) = cells aliquoted at early log phase of growth; (**B**,**D**) = cells aliquoted at stationary phase. Scale = 10 µm.

Epifluorescence images taken during the early log and stationary growth phases are shown in Figure 4A–D for each DAPI-stained bacterial strain. Owing to the shift in the emission wavelength from 475 to 525–550 nm, resulting from the binding of DAPI to polyP, polyP appears as distinct yellowish-green inclusions (polyP-DAPI complexes) that are clearly distinguishable from the cells that appear blue (DNA-DAPI complexes). *S. mutans* did not exhibit polyP inclusions during any phase of its growth cycle (Figure 4A,B). However, distinct polyP inclusions were observed in *C. matruchotii* (Figure 4C,D) in both the early log and stationary phases of growth.

## 4. Discussion

*C. matruchotii* sequestered available P_i_ from the surrounding medium, and this preceded the synthesis and accumulation of polyP inclusions, as observed in the epifluorescence images. Our results suggest that P_i_ accumulation does not contribute to the pathogenicity of *S. mutans* under the conditions studied here in the preferred oxygen tension of anaerobiosis. *S. mutans* did not accumulate polyP inclusion bodies, nor did it take up high amounts of P_i_. However, *S. mutans* and *C. matruchotii* grew similarly in BHI broth media. We suggest that *C. matruchotii* accumulates P_i_ beyond its normal growth requirements, which is commonly associated with polyP accumulation and is referred to as “luxury P_i_ uptake” [39,40]. This luxury uptake of aqueous phase P_i_ has been found elsewhere in nature, especially in marine environments where apatite solubility is influenced by aquatic bacteria that take up P_i_ in order to synthesize and accumulate polyP [41,42,43]. These long polymers of polyP provide bacteria with several competitive advantages by serving as reservoirs of P_i_ and metabolic energy, but can also be linked to gene regulation, stress response, and cell survival [3,44]. The justification for testing *S. mutans* for polyP accumulation may not be immediately apparent, because *S. mutans* lack the genes that encode the canonical proteins associated with polyP metabolism, polyphosphate kinases, and exopolyphosphatase. However, since bacteria are thought to be capable of accumulating polyP by other unknown mechanism(s) [45], our experiment was necessary to examine the ability of *S. mutans* to accumulate polyP or sequester P_i_ by other means. Historically, *S. mutans* was investigated as one of the sole causative bacteria in dental caries [46,47]. The lack of potential to accumulate polyP may explain why P_i_ accumulation and polyP synthesis was never investigated extensively, given the historical significance of *S. mutans* in cariology [48].

While it is well known that free P_i_ is liberated during tooth demineralization from the acid generation produced by *S. mutans*, this study suggests that *S. mutans* may primarily derive phosphorus from organic nutrients (as opposed to inorganic phosphate). Studies have shown that carbon sources such as glucose, and inorganic nitrogen, as well as increased concentrations of yeast extract (up to 0.5 g/L), can influence organic phosphorus solubilization by bacteria [31]. This may explain the increase in P_i_ (by approximately 1.0 mM) observed in the stationary phase of growth in *S. mutans* cultures. Our results led us to develop a working hypothesis for future testing, postulating that P_i_ solubilized from organic sources by *S. mutans* has the potential to accumulate by other neighboring bacteria within the oral environment.

The possible ecological advantages of oral bacteria accumulating polyP are unknown, although the many benefits of polyP accumulation in non-oral taxa have been well established. Among other advantages, polyP inclusions in non-oral taxa serve as a long-term stable energy reserve and can improve the coordination of stress response conditions [49]. In vitro polyP accumulation should be viewed in the context of in situ experiments that have measured substantial decreases in calcium and phosphate concentrations within dental plaque during exposure to saccharides [50].

In situ experiments have not identified the mechanisms associated with this ion drawdown in plaque, but our work encourages the examination of polyP-associated mechanisms [30], and future studies can quantitatively examine P_i_ uptake per bacterial cell of oral biofilms. *C. matruchotii* has been associated clinically, and in model systems, with mineralization processes [51,52,53,54,55], but the underlying mechanisms behind these associations are unknown, and studies have predominantly focused on Ca^2+^. Some studies have shown that, compared to *S. mutans*, *C. matruchotii* and other Streptococcus species are capable of promoting the formation of hydroxyapatite-like crystals when grown in calcium-enriched media [56,57,58]. Yet, no significant Ca^2+^ uptake (*p* value < 0.05) was observed under our test conditions. The reason for the differences observed between P_i_ and Ca^2+^ cellular uptake in the late stationary phase is possibility related to the critical importance of P_i_ in growth and survival [59]. In well-characterized bacterial models, P_i_ concentrations are 1–2 orders magnitude higher than Ca^2+^, and P_i_ is crucial in membrane integrity, protein signaling, and nucleic acid synthesis [60,61]. While the negatively charged polyP anion can complex with calcium cations and form complexes, many other monovalent and divalent ions (Na^+^, K^+^, Mg^2+^) can also act as counter ions to the negatively charged phosphate residues within polyP [62]. Calcium does not need to be internalized exclusively, in a 1:1 ratio to P_i_, to stabilize the negatively charged PolyP phosphate residues. While the reduction in Ca^2+^ concentration was not statistically significant, it is important to recognize that sequestering P_i_ as polyP within the cell and changing the external P_i_ concentration can influence the solubility of HA at the tooth surface by pushing the reaction of HA solubility towards undersaturated conditions, thereby favoring demineralization.

The study has several limitations. The study compared a model organism that possessed common polyP metabolic genes (*C. matruchotii*) with another bacterium (*S. mutans*) that did not possess these genes. This work does not imply that the magnitude of P_i_ uptake will occur under similar environmental conditions in other bacterial species where polyP genes are present or absent. Future studies are needed to understand single- and multi-species bacterial models under several different types of environmental conditions, such as dissolved oxygen and P_i_ concentration fluctuations. While *C. matruchotii* has been associated with caries-free activity [59], its association and prevalence within the microbiome in caries-active versus caries-free individuals is equivocal [10]. The role of *C. matruchotii* in caries activity or the attenuation of caries therapy via P_i_ may depend on unidentified factors such as an alkaline phosphatase interaction with external polyP after cell death.

In summary, *C. matruchotii* removed P_i_ from the medium and accumulated P_i_ as polyP inclusions. *S. mutans*, which is traditionally accepted as a highly cariogenic microorganism, did not have detectable polyP inclusions or a notable uptake of P_i_. This study may have implications for the development of topical therapies and bioactive dental materials that aim to inhibit demineralization. The efficacy of these technologies may be attenuated by the possible role of specific oral bacteria by ‘capturing’ P_i_ before the ion is able to be incorporated into the tooth structure. While our work does not directly test this hypothesis, it demonstrates that future in vitro studies should investigate if *C. matruchotii* and other bacteria with polyP biosynthetic genes influence the short- and long-term efficacy of bioactive materials and topical therapies that provide ions for demineralization inhibition.

## Data Availability

The original contributions presented in this study are included in the article. Further inquiries can be directed to the corresponding author.
